# Data characterizing the biophysical and nitric oxide release properties of the tDodSNO – Styrene maleic anhydride nanoparticle SMA-tDodSNO

**DOI:** 10.1016/j.dib.2018.10.149

**Published:** 2018-11-03

**Authors:** Houman Alimoradi, Anita Barzegar-Fallah, Ivan A. Sammut, Khaled Greish, Gregory I. Giles

**Affiliations:** a Department of Pharmacology and Toxicology, University of Otago, Dunedin, New Zealand; b College of Medicine and Medical Sciences, Department of Molecular Medicine and Nanomedicine Unit, Princess Al-Jawhara Center for Molecular Medicine and Inherited Disorders, Arabian Gulf University, Manama, Bahrain,

## Abstract

Nitric oxide (NO) donor drugs have a range of clinical applications, and are also being developed as therapeutics for the potential treatment of multiple diseases. This article presents data describing the synthesis and characterisation of a novel NO releasing nanoparticle formed by encapsulation of the NO donor tDodSNO into a co-polymer of styrene and maleic acid (SMA) to afford SMA-tDodSNO. The pharmacological activity of SMA-tDodSNO is discussed in our accompanying manuscript “Encapsulation of tDodSNO generates a photoactivated nitric oxide releasing nanoparticle for localized control of vasodilation and vascular hyperpermeability”. (Alimoradio et al. [1]).

**Specifications table**

**Table:** 

Subject area	*Pharmacology*
More specific subject area	*Drug delivery*
Type of data	*Table, photographs, figures, graphs, electronic spectra.*
How data were acquired	*HPLC, Jenway 6715 UV–vis spectrometer, Malvern Zetasizer ZEN3600.*
Data format	*Analyzed.*
Experimental factors	*For NO release, SMA-tDodSNO was photoactivated using a cold light source.*
Experimental features	*Data reporting the loading of tDodSNO into SMA-tDodSNO; dynamic light scattering graphs showing the size and charge of the SMA-tDodSNO nanoparticles; photographs demonstrating the improved aqueous solubility of SMA-tDodSNO vs tDodSNO; electronic spectra characterizing NO release from SMA-tDodSNO.*
Data source location	*University of Otago, Dunedin, New Zealand.*
Data accessibility	*Data provided in article.*
Related research article	*Alimoradi, H., Barzegar-Fallah, A., Sammut, I.A., Griesh, K., and Giles, G.I. Encapsulation of tDodSNO Generates a Photoactivated Nitric Oxide Releasing Nanoparticle for Localized Control of Vasodilation and Vascular Hyperpermeability. Free Radic Biol Med, 2018, DOI: 10.1016/j.freeradbiomed.2018.10.433**[1]*.

**Value of the data**

• The data describe the synthesis, biophysical, and NO releasing characteristics of the novel nanoparticle SMA-tDodSNO, which can be used in further studies to explore the therapeutic potential of NO releasing drugs.
• The methodology described can be applied to generate and characterize new NO releasing nanoparticles.
• The NO release characteristics of SMA-tDodSNO can be used as a comparison to evaluate the activity of new NO donors.

## 1 Data

Following chemical synthesis, the yield of SMA-tDodSNO was obtained via weighing the product nanoparticle. The amount of tDodSNO in SMA-tDodSNO was then obtained by HPLC analysis, and nanoparticle loadings calculated according to theoretical yield calculations. SMA-tDodSNO solubility data were visually confirmed via photographs of tDodSNO and SMA-tDodSNO solutions. Nanoparticle biophysical characteristics were quantified by dynamic and electrophoretic light scattering of SMA-tDodSNO in deionized water. NO release from SMA-tDodSNO was measured by monitoring the oxidation of the protein oxymyoglobin via ultraviolet-visible spectroscopy, and analysis of the resulting spectra.

## 2 Experimental design, materials and methods

### 2.1 Synthesis of tDodSNO

tDodSNO was synthesized as previously described [2]. As a typical procedure, 2 ml of tert-dodecylmercaptan (11.32 mmol) were dissolved in 10 ml dry dichloromethane, and 1.5 ml of tert-butyl nitrite (11.33 mmol) added dropwise at 0 °C under argon. The mixture was stirred for 30 min, and then allowed to warm to ambient temperature and stirred for a further 30 min. The resulting mixture was evaporated under reduced pressure at 35 °C to obtain a dark green oil. The crude product was flash chromatographed on silica gel eluting with dichloromethane/hexane (3:97 v/v) to afford tDodSNO (2.48 g, 95% yield). Characteristic spectra were as previously reported [2].

### 2.2 Encapsulation of tDodSNO into SMA nanoparticles

Poly(styrene-co-maleic anhydride), SMA, was supplied by Sigma-Aldrich (St Louis, MO, USA). The co-polymer was cumene terminated, with a styrene:maleic anhydride feed ratio of 3:1, and an average Mn ~1600. To generate SMA-tDodSNO, nanoparticle formation was initiated using a previous method that has generic applicability for drug encapsulation within SMA [3]. Initially a SMA solution (10 mg/ml) was prepared by solubilizing 1 g of SMA powder in 100 ml of 1 M NaOH at 70 °C for 3 h. A 3.75 ml aliquot of this solution was cooled to 40 °C, and then acidified to pH 5.0 by the addition of 1 M HCl. tDodSNO (12.5 mg in 2 ml DMSO) was then added dropwise under continuous stirring (1300 rpm) to afford a cloudy solution, followed by the addition of 1-ethyl-3-(3-dimethylaminopropyl)carbodiimide (37.5 mg in 2 ml ddH_2_O). After stirring for 10 min, a further 10 ml ddH_2_O was added, the solution stirred for an additional 2 min, and the pH increased to 11 by the addition of 1 M NaOH. Once a clear green solution was obtained the pH was adjusted to 7.4 with 1 M HCl, and the resultant NP purified by 4 cycles of ultrafiltration with deionized water using an ultrafiltration system (Merck Millipore, Auckland, NZ) with a 10 kDa XL filter (Pellicon, Merck Millipore, Auckland, NZ), followed by lyophilization to afford a light green powder. To measure tDodSNO loading, known weights of SMA-tDodSNO were dissolved in methanol, and the concentration of tDodSNO quantified using HPLC [1]. tDodSNO loading was expressed as a w/w % of tDodSNO:SMA (Table 1).

**Table 1:** SMA-tDodSNO loading.

**SMA-tDodSNO theoretical maximum yield (mg)**	**SMA-tDodSNO experimental yield (mg)**	**Recovery (%)**	**tDodSNO loading (%, w/w)**
200	120.6	60.3	20.1
200	130.2	65.1	22.6
200	156.1	78.0	20.4
**Mean±SD**	135.6 ± 18.4	67.8 ± 9.1	21.1 ± 1.4

### 2.3 Biophysical characterization of SMA-tDodSNO

Nanoparticle size and zeta potential were established by dynamic and electrophoretic light scattering of solutions of the nanoparticle in deionized water at room temperature using a Malvern Zetasizer ZEN3600 (Malvern Instruments Inc., Westborough, MA, USA). All measurements were repeated in triplicate.

**Fig. 1:**
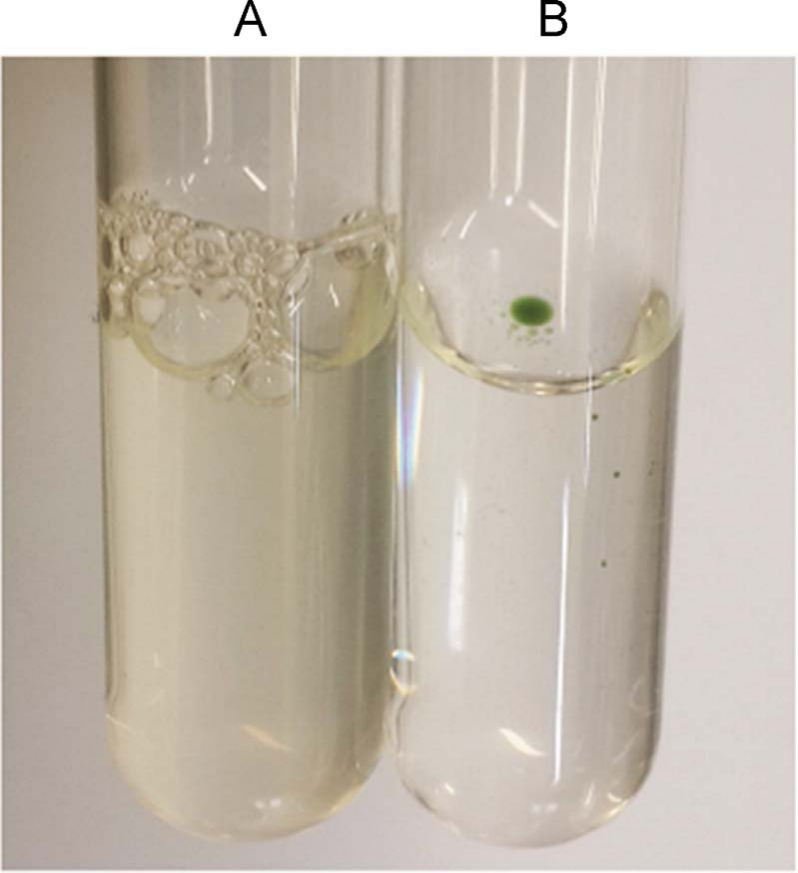
Solubility comparison between SMA-tDodSNO and tDodSNO. (A) SMA-tDodSNO (1 mM) in PBS. (B) tDodSNO (1 mM) in PBS.

**Fig. 2:**
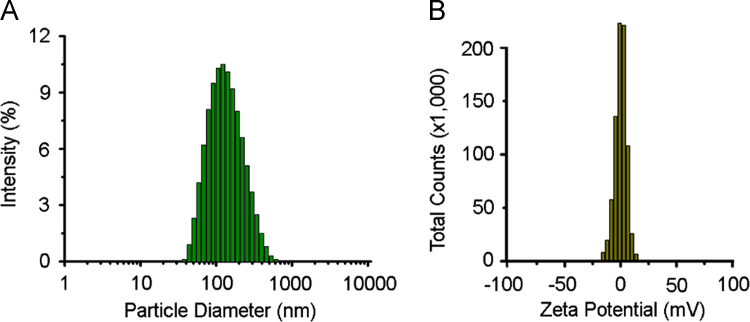
Biophysical characteristics of SMA-tDodSNO. (A) Particle size distribution. (B) Zeta potential.

SMA-tDodSNO fully dissolved in PBS at pH 7.4, while tDodSNO partitioned into an immiscible green layer (Fig. 1). The nanoparticle had a mean diameter of 230 ± 40 nm, with a polydispersity index of 0.21 ± 0.02 (Fig. 2). The surface charge of SMA-tDodSNO in ddH_2_O was essentially neutral, with a mean Z-potential of -0.001 ± 0.032 mV (Fig. 2).

### 2.4 Quantification of NO release

**Fig. 3:**
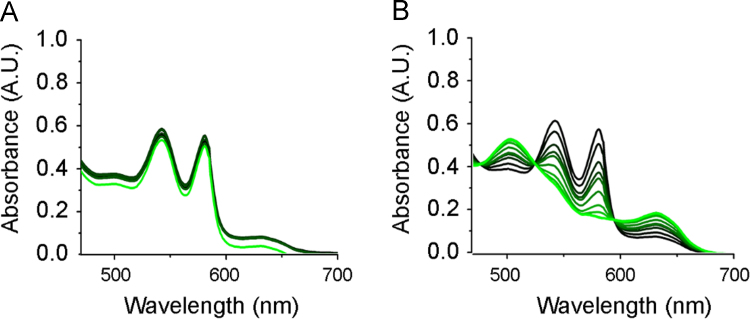
Quantification of NO release from SMA-tDodSNO. SMA-tDodSNO (50 µM) was added to MbO_2_ (50 µM) in PBS on ice, and electronic spectra of MbO_2_ acquired over 30 min. A: Control conditions (no photoactivation), B: Photoactivation (2700 W/m^2^). Overlaid spectra indicate the time course (black to green over 30 min) and the extent of oxidation of MbO_2_ to met-myoglobin.

Horse heart myoglobin was dissolved in phosphate buffer (pH 7.4), and then reduced by the addition of excess sodium dithionite for 5 min. The mixture was passed through a PD10 desalting column (Sephadex G-25M, GE Healthcare, Auckland, NZ) to purify and oxygenate the reduced myoglobin to yield oxymyoglobin (MbO_2_). MbO_2_ concentration was determined by its electronic absorption at *λ* = 542 nm (*ε* = 13,900 M^−1^ cm ^−1^) [4]. As MbO_2_ is sensitive to light [5], experiments were performed on ice. NO release from SMA-tDodSNO was quantified as previously described [6] using a cold light source for photoactivation [1] (Fig. 3).
