# Supplementary material for: Data characterizing the biophysical and nitric oxide release properties of the tDodSNO – Styrene maleic anhydride nanoparticle SMA-tDodSNO
Source: Data Brief. 2018 Nov 3;21:1771–5. doi: 10.1016/j.dib.2018.10.149 (PMC6249518; doi:10.1016/j.dib.2018.10.149)
Supplement: Supplementary file 1 — Supplementary material [file mmc1.docx]

The authors declare no interests
